# Cases of Hospital Gangrene Treated in Church U. S. A. Military General Hospital, during the Month of August, 1863

**Published:** 1864-01

**Authors:** 


### CASES OF HOSPITAL GANGRENE

TREATED IN CHURCH U. S. A. MILITARY GENERAL HOSPITAL, DURING THE MONTH OF AUGUST, 1863.

[For Table, see next two pages.]

### REMARKS.

1. Died of Pyæmia. Was moribund when admitted.
2. Transferred to Jackson Hospital, August 17th, 1863
3. " " " " " "
4. Now under treatment, wounds filling up.
5. Was infected when admitted. Sepsis and Thrombi formed on autopsy.
6. Transferred to Jackson Hospital, August 8th, 1863.
7. " " " " 4th, "
8. Lower Condyle of humerous is necrosed.
9. Transferred to Jackson Hospital, August 19th, 1863
10. Died of Pyæmia.
11. Thrombus in aorta. had Sepsis when admitted.
12. Died of Mechanical Pneumonia and Thrombus.
13. Transferred to Overton Hospital, August 24th, 1863
14. Under treatment now. Tendo Achilles involved.
15. Transferred to Overton Hospital, August 19th, 1863.
16. " " " " 21st, "
17. Still under treatment, had facial Erysipelas, August 8th, 1863.
18. Died of Diarrhœa. Wound was granulating since August 11th, 1863.
19. Transferred to Overton Hospital, August 5th.
20. " " " " 21st. Deep pereneal fascias sloughed or come away.
21. Mechanical Pneumonia and Sepsis when admitted
22. Pyæmia, had infection when admitted. Wound did not granulate.
23. Transferred to Union Hospital, August 4th.
24. " " " " 5th.
25. Still under treatment.
26. Admitted 1st August. Moribund. Died the 2d, of Secondary Hemorrhage.
27. Reappeared Aug. 19, wound was granulating from 13th to 19th of Aug.
28. Died of Pyæmia.
29. Transferred to Gayoso Hospital, August 19th.
30. Reappeared Aug. 19th, had no bromine, used nitric acid—will die.
31. Secondary hemorrhage in the night.
32. Transferred to Adams Hospital, August 17th.
33. " Webster " " 21st.
34. Still under treatment.
35. Bromine not applied to all, was burrowing. Died from Thrombus in left Auricle weighing 482 grains and occluded auriculo ventricular opening.
36. Transferred to Webster Hospital, August 23d.
37. Still under treatment.
38. " "
39. " " His condition is doubtful.
40. " "
41. " "
42. " "
43. " " Same as No. 24, relapsed and returned.
44. " " Ball removed from tibia, was in cancellus structure.
45. " "
46. " " Has Sepsis.

Compiled by Surgeon GEO. R. WEEKS, U. S. A.

### Cases of Hospital Gangrene treated in Church U. S. A.

| No | Name. | Rank | Regiment. | Co· | Where wounded· | When. | Tissues Involved | region of wound. |
|---|---|---|---|---|---|---|---|---|
| 1 | Miller, Jos. T.... | priv. | 33d Iowa | F | .......... | ...... | mus integ cel & bone | right thigh. |
| 2 | Cooms, Reuben.. | corp | " " | C | Helena, Ark | July 4 | | dorsal region. |
| 3 | Nelson, John.... | priv | 6th Mo | D | Cham'n Hill | May 16 | | left heel. |
| 4 | Graham, Sandf'rd | " | 33d Iowa | C | Helena, Ark | July 4 | | left arm & side |
| 5 | Johnson, Oliver.. | " | " " | C | " " | " | | right elbow. |
| 6 | Arbuthnot, Saml. | " | 28th " | F | " " | " | | left hand. |
| 7 | Louis, Fredrick .. | " | 1 Ind Cav | F | .......... | ...... | | right hand. |
| 8 | Whitaker, John.. | serg. | 33d Iowa | E | Helena, Ark | July 4 | | left elbow joint |
| 9 | Pfister, George... | priv. | 48th Ohio | C | Vicksburg | May 22 | knee joint | left knee. |
| 10 | Hughes, Elijah... | " | 4th Va. | I | " | May 19 | | left loin. |
| 11 | Beauer, Jas. A... | " | 33d Iowa | A | Helena, Ark | July 4 | | right instep. |
| 12 | Frank, John..... | " | 6th Mo. | F | Vicksburg | July 15 | mus integ and bone. | right shoulder. |
| 13 | Oxford, John.... | " | 3rd Ark. | K | Helena, Ark | July 4 | | right thigh. |
| 14 | Dorton, Lafayette | " | 9th Mo. | B | " | " | | right calf. |
| 15 | Riggs, T. D...... | " | Ha'thorns | I | " | " | | left foot. |
| 16 | Coulter, B. F.... | corp | Bell's Ark | K | " | " | | right heel. |
| 17 | Bryon, Robert... | priv. | 8th " | D | " | " | | left thigh. |
| 18 | Gray, John...... | " | 2d " | C | " | " | | left leg. |
| 19 | Twitnan, D. L... | " | 8th Mo. | I | .......... | ...... | | right thigh. |
| 20 | Harris, W. H.... | " | 33d Iowa | C | Helena, Ark | July 4 | | right thigh. |
| 21 | Butumbaugh, M.. | " | 99th Ill. | C | Vicksburg | May 22 | | left thigh. |
| 22 | Wilkie, August.. | serg. | 33rd Mo. | H | Helena, Ark | July 4 | | right knee. |
| 23 | Graham, Robert.. | " | " " | E | " | " | | right arm. |
| 24 | Goff, William.... | priv. | 33d Iowa | I | " | " | | right leg. |
| 25 | Alt, Michael..... | " | 26th Mo. | F | Cham'n Hill | May 16 | | right leg. |
| 26 | Ijames, V. M..... | " | Harts Ark | — | " " | ...... | | left dorsum. |
| 27 | Cole, G. A....... | " | 7th Mo. | K | Helena, Ark | July 4 | | left ankle. |
| 28 | Alexander, John. | " | 33d Iowa | E | .......... | ...... | | loin. |
| 29 | Holzimu, Wm... | " | 12th Mo. | H | Vicksburg | May 22 | | left thigh. |
| 30 | Sullard, J. A.... | " | 7th Mo. | A | Officers Hos | Aug 1 | | right ankle. |
| 31 | Campbell, John A | corp | 81st Ill. | H | Vicksburg | May 22 | | left hand. |
| 32 | Fuller, S. W..... | " | 26th Con. | A | Port Huds'n | Jun 14 | | right ankle. |
| 33 | McEvoy, James.. | priv. | 7th Mo. | D | Raymond | May 12 | | right arm. |
| 34 | Cloud, Allen..... | " | 22d Iowa | H | Vicksburg | May 22 | | left arm. |
| 35 | Millceps, C. F... | " | 18th Ark. | G | Port Huds'n | May 27 | | right thigh. |
| 36 | McClure, Samuel. | " | 83d Ind. | H | Vicksburg | May 19 | | stump left leg. |
| 37 | Butler, John M.. | " | 5th Iowa. | D | Cham'n Hill | May 16 | | stump rightleg. |
| 38 | Onert, Peter..... | " | 124th Ill. | F | " " | May 16 | | left thigh. |
| 39 | Goodrich, I...... | " | 5th Mo. | E | Helena, Ark | July 4 | | gential & dorsil |
| 40 | Waters, A. M.... | " | Bell's Ark | E | " | " | | left thigh. |
| 41 | Scott, James.... | " | 1st Mo. | A | " | " | | right thigh. |
| 42 | Watts, L. L.... | " | 1st Ark. | D | " | " | | left shouider. |
| 43 | Goff, William.... | " | 33d Iowa | I | " | " | | right leg. |
| 44 | Brickerhoff, B.,, | " | 47th Ohio | K | Vicksburg | May 19 | tibia | left leg. |
| 45 | Grant, F. M...... | " | 3rd Iowa | E | " | Jun 22 | | right calf. |
| 46 | Witby, T. T..... | " | 29th Iowa | F | Helena, Ark | July 4 | bone | stump ri't thigh |

### Military General Hospital, during the month of Aug. 1863.

| When Gangrene began. | When admitted | When arrested. | Duration in this Hospital. | Local Treatment. | Gen'al Treatment. | Died. | Convalesced. |
|---|---|---|---|---|---|---|---|
| ...... | July 30 | ........ | ...... | chlo. soda. | Tonics, skin and nut. diet. | Aug 8 | ... .... |
| July 28 | " " | Aug 10 | 1 day | bromine, Aug. 9 | | ...... | convalesced |
| " 25 | " " | " 12 | 2 " | " " 10 | | ...... | " |
| " 10 | " " | " 11 | 1 " | " " 9 | | ..... | " |
| " 10 | " " | " 12 | 2 " | " " 10 | | Aug 13 | .......... |
| ...... | " " | ........ | ...... | chlo. soda. | | ...... | convalesced |
| ...... | " " | Aug 3 | ...... | sul zinci. chlo. soda. | | ...... | " |
| Jul 19 | " " | " 10 | 1 day | bromine, Aug. 9 | | ...... | " |
| " 25 | " " | " 10 | 1 " | " " 10 | | ...... | " |
| Jun 27 | " " | not arres' | ...... | nit acid and nit aly. | | Aug 2 | .......... |
| July 17 | " " | Aug 12 | 3 days | bromine, 9, 10, 11 Aug | | " 18 | .......... |
| " 20 | " " | " 6 | ...... | nit acid and chlo. soda | | " 8 | .......... |
| " 20 | " 31 | " 11 | 1 day | bromine, Aug. 10 | | ...... | convalesced |
| " 10 | " " | " 13 | 2 " | " " 11 | | ...... | " |
| " 12 | " " | " 10 | 1 " | " " 9 | | ...... | " |
| " 24 | " " | " 12 | 2 " | " " 11 | | ...... | " |
| " 28 | " " | " 13 | 2 " | " " 10 | | ...... | " |
| " 22 | " " | " 11 | 1 " | .................. | | Aug 23 | .......... |
| ...... | " " | ........ | ...... | bromine, Aug. 9 | | ...... | convalesced |
| July 20 | " " | Aug 10 | 1 day | " " 9 | | ...... | " |
| " 27 | " " | " 10 | 1 " | " " 10, 11 | | Aug 14 | ..... .... |
| " 10 | " " | " 12 | 2 " | chlo. soda. | | " 17 | .......... |
| ...... | " " | ....... | ...... | chlor'te soda & nit acid | | ...... | convalesced |
| ..... | Aug 1 | Aug 5 | 1 day | bromine, Aug. 10 | | ...... | " |
| July 22 | " " | " 11 | ...... | per sul. ferri. | | ...... | " |
| ..... | " " | ........ | 4 days | bromine, Aug. 9 | | Aug 2 | ... ..... |
| July 25 | " 2 | Aug 13 | ...... | nit. acid and chlo.soda | | " 21 | .......... |
| ...... | " " | .... ... | 3 days | bromine, Aug. 9 | | " 7 | ......... |
| July 22 | " " | Aug 12 | 4 " | " " 9, 12 | | .... | convalesced |
| Aug 1 | " 5 | " 13 | 4 " | " " 8,10,11,12 | | ...... | .......... |
| May 30 | " 8 | " 12 | 1 " | " " 10 | | Aug 13 | .......... |
| July 25 | " 9 | " 11 | 1 " | " " 10 | | ...... | convalesced |
| " 20 | " 10 | " 11 | 7 " | com sol bro fro 13 to 20 | | ...... | " |
| Aug 4 | " 13 | " 20 | ... .. | " 14 15 | | ...... | " |
| " 8 | " 14 | ........ | 2 days | " Aug. 16 | | Aug 27 | .......... |
| " 8 | " 15 | Aug 18 | 4 " | " " 16 | | ...... | convalesced |
| July 20 | " 15 | " 20 | 1 " | " " 28 | | ...... | " |
| Aug 1 | " 22 | " 28 | .... | " " 26 | | .. .. | " |
| " 20 | " 24 | wo'nd bet | 2 days | " " 25 | | ...... | .......... |
| " 10 | " 24 | Aug 27 | 1 " | " " 25 | | ...... | convalesced |
| " 4 | " 24 | " 26 | 1 " | " " 25 | | ...... | " |
| " 10 | " 24 | " 26 | 1 " | " " 27 | | ...... | " |
| " 15 | " 26 | " 28 | ...... | " " 28 | | ...... | .......... |
| " 15 | " 26 | look bet. | ...... | " " 28 | | ...... | .......... |
| " 16 | " 28 | " " | ..... | " " 28 | | ...... | .. ....... |
| " 1 | " 29 | " " | ....... | " " 28 | | ...... | .......... |

